# New Directions in Cariology Research

**DOI:** 10.1155/2010/525417

**Published:** 2010-08-19

**Authors:** Alexandre R. Vieira

**Affiliations:** Department of Oral Biology, School of Dental Medicine, University of Pittsburgh, Pittsburgh, PA 15261, USA

Although there has been more than 100 years since Miller first postulated about the caries etiopathogenesis, this disease remains the most prevalent noncontagious infectious disease in humans. It is clear that the current approaches to decrease the prevalence of caries in human populations, including water fluoridation and school-based programs, are not enough to protect everyone. The US National Institutes of Health Consensus Development Program released a statement in 2001 entitled “Diagnosis and Management of Dental Caries Throughout Life: (NIH Consensus statement Online 2001 March 26–28; 18(1): 1–24) and listed six major clinical caries research directions.

- The “epidemiology of primary and secondary caries” needs to be systematically studied with population cohort studies that collect information on natural history, treatment, and outcomes across the age spectrum.
- Research into “diagnostic methods”, including established and new devices and techniques, is needed. Development of standardized methods of calibrating examiners is also needed.
- “Clinical trials” of established and new treatment methods are needed. These should conform to contemporary standards of design, implementation, analysis, and reporting. They should include trials of efficacy.
- Systematic research on caries “risk assessment” is needed using population-based cohort techniques.
- Studies of “clinical practice” including effectiveness, quality of care, outcomes, health-related quality of life, and appropriateness of care are needed.
- “Genetic” studies are necessary to identify genes and genetic markers of diagnostic, prognostic, and therapeutic value.

This Special Issue is a sample of the current research efforts addressing a subset of the topics described above. I would like to thank the authors for their excellent contributions, in addition to many colleagues who assisted me in the peer-review process. Finally, I would like to thank the support provided by my Guest Editors, Dr. Marilia Buzalaf, from the University of São Paulo, Brazil, and Dr. Figen Seymen, from the Istanbul University, Turkey.

On the topic of “epidemiology and caries risk assessment” F. J. S. Pieralisi et al., K. K. Mäkinen, J. Vanobbergen et al., M. Okada et al., M. Ferraro and A. K. Vieira, A. P. Dasanayake et al., and J. D. Ruby et al. provide different perspectives to the problem which are good examples of how difficult is to study this multifaceted disease in a more comprehensive fashion.

There is currently great interest in “diagnostic methods,” and the use of technology to differentiate diseased and healthy tissue, as well as to provide care. Different aspects of diagnostic and treatment approaches are addressed by S. Umemori et al., M. Aspiras et al., J. Wu et al., L. Karlsson, M. Yildirim et al., and A. Huminicki et al.

The biggest challenge continues to be designing rigorous clinical trials that can provide conclusive answers related to approaches that more effectively control caries at the segments of the population with higher risk for developing the disease.

I invite you to read, evaluate, and share this collection of 13 papers that comprise this special issue. Furthermore, I hope the readers will be interested in participating more actively in this debate of what approaches are more efficient to revert the current figures of caries prevalence and what aspects of this disease should be the focus of research in the coming years.

*Alexandre R. Vieira* *Alexandre R. Vieira*

